# Motorboat noise impacts parental behaviour and offspring survival in a reef fish

**DOI:** 10.1098/rspb.2017.0143

**Published:** 2017-06-07

**Authors:** Sophie L. Nedelec, Andrew N. Radford, Leanne Pearl, Brendan Nedelec, Mark I. McCormick, Mark G. Meekan, Stephen D. Simpson

**Affiliations:** 1 School of Biological Sciences, University of Bristol, Life Sciences Building, 24 Tyndall Avenue, Bristol BS8 1TQ, UK; 2 Ecology and Evolutionary Biology, University of Colorado Boulder, 1900 Pleasant Drive, 334 UCB, Boulder, CO 80309-0334, USA; 3 School of Marine and Tropical Biology, James Cook University, Townsville, Queensland 4811, Australia; 4 Australian Institute of Marine Science, The University of Western Australia (MO96), 35 Stirling Highway, Crawley WA 6009; 5 Biosciences, College of Life and Environmental Sciences, University of Exeter, Stocker Road, Exeter EX4 4QD, UK

**Keywords:** anthropogenic noise, parental care, pollution, Lizard Island, motorboat-noise playback

## Abstract

Anthropogenic noise is a pollutant of international concern, with mounting evidence of disturbance and impacts on animal behaviour and physiology. However, empirical studies measuring survival consequences are rare. We use a field experiment to investigate how repeated motorboat-noise playback affects parental behaviour and offspring survival in the spiny chromis (*Acanthochromis polyacanthus*), a brooding coral reef fish. Repeated observations were made for 12 days at 38 natural nests with broods of young. Exposure to motorboat-noise playback compared to ambient-sound playback increased defensive acts, and reduced both feeding and offspring interactions by brood-guarding males. Anthropogenic noise did not affect the growth of developing offspring, but reduced the likelihood of offspring survival; while offspring survived at all 19 nests exposed to ambient-sound playback, six of the 19 nests exposed to motorboat-noise playback suffered complete brood mortality. Our study, providing field-based experimental evidence of the consequences of anthropogenic noise, suggests potential fitness consequences of this global pollutant.

## 1. Introduction

Mounting evidence indicates that anthropogenic noise, a pervasive pollutant, disturbs and has detrimental effects on a wide range of species, including mammals, birds, anurans, fishes, and invertebrates (see reviews in [1–6]). Studies showing short-term behavioural and physiological impacts of noise are numerous [7–10]. Some chronic effects of noise, such as altered habitat use and reduced pairing success, have also been identified [11–12]. However, studies that reveal impacts on reproduction or survival via experimental manipulations, with suitable controls and replicates are rare (for exceptions, see [12–14]).

Anthropogenic noise has been shown to affect parental behaviour, including feeding, nest maintenance, and defence. Specific examples include reduced time spent tending nests in the damselfish *Chromis chromis* [15], increased latency to visit a nest-box in great tits (*Parus major* [9]), and increased missed detections of parents leading to reduced begging in tree swallows (*Tachycineta bicolor* [16]). While noise has clear effects on parental-care behaviour in the short term, there remains the possibility that ongoing exposure would allow animals to habituate, compensate, or move away from the source [4,5,17,18]. Therefore, longer-term studies considering offspring survival as well as parental care are required.

We investigated the effects of repeated exposure to anthropogenic noise on male parental behaviour, and offspring growth and survival in a coral reef fish, the spiny chromis (*Acanthochromis polyacanthus*). We used playbacks of recordings of motorboat noise, since that is the most common source of anthropogenic noise in shallow reef environments [19]. *A. polyacanthus* exhibits bi-parental care of eggs and larvae at nests within shallow reef habitat in the tropical Western Pacific [20,21]; males contribute more care than females in this species (MI McCormick 2013, personal observation). One of the most vital roles of adults is to guard their brood by chasing away potential predators and competitors [22]. Parental care is energetically expensive [23] and thus it is important that parents feed regularly. Moreover, *A. polyacanthus* parents provide their offspring with mucus, which can contain proteins, hormones, ions, microorganisms, immunoglobulins, and secretocytes undergoing mitosis [24–26]. Mucus is delivered via ‘glancing’ (also called ‘parent-touching’ or ‘contacting’ in other species); parents are relatively passive in this process, but do actively avoid offspring on some occasions. These three key parental-care behaviours (guarding, feeding, and glancing) are all easily observed in *A. polyacanthus* in its natural habitat [27,28].

We exposed 38 *A. polyacanthus* nests with recently hatched juveniles to 12 days of playback of either motorboat passes recorded near reefs or natural ambient sound recorded at the same locations. We collected data throughout the acoustic-exposure period to answer three main questions. (i) Is guarding, feeding, and glancing behaviour of brood-guarding males negatively impacted by the addition of motorboat noise? (ii) Can an increased frequency of defensive acts by brood-guarding males be explained by changes in the prevalence or behaviour of other local species? (iii) Do *A. polyacanthus* offspring at nests experiencing motorboat-noise playback suffer reduced growth or survival compared to control nests with playback of ambient reef sound?

## 2. Methods

### (a) General experimental set-up

**Figure 1.**
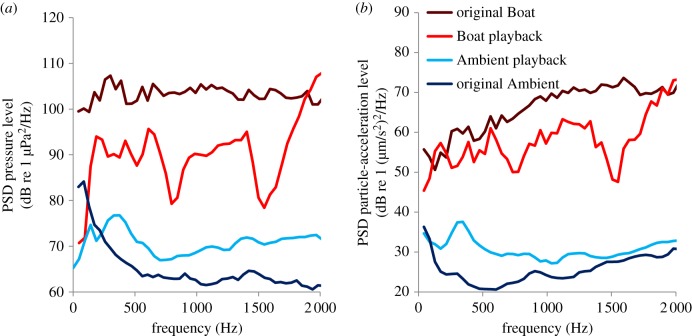
(*a*) Power spectral density (PSD) for sound-pressure levels and (*b*) *x*-axis particle-acceleration levels. Original recordings of motorboat noise and ambient sound are compared with playbacks of these recordings at experimental sites (mean of 60 s samples, window length = 1 024, overlap = 50%). Sound-pressure playbacks were recorded at 19 Boat and 19 Ambient sites while particle acceleration could only be recorded at one Boat and one Ambient site. Playbacks reveal a peak in sound level around 2 000 Hz and troughs around 800 and 1 500 Hz (artefacts of the loudspeakers used), but for both sound pressure and particle acceleration, motorboat noise and motorboat-noise playbacks were louder than ambient sound and ambient-sound playbacks at all sites, at frequencies produced by the speaker (more than 100 Hz). Also, real motorboats were louder than motorboat-noise playbacks, but real ambient sound was quieter than ambient-sound playbacks, making our experimental playback levels a conservative representation of reality. (Online version in colour.)

Data were collected between October and December 2013 at Lizard Island Research Station (14°4′S 145°28′E), Great Barrier Reef, Australia. Thirty-eight *A. polyacanthus* nests with new clutches of juveniles were studied; full details in electronic supplementary material. Half of the nests were allocated to the ‘Ambient’ and half to the ‘Boat’ sound treatment. Four replicate playbacks were constructed for each treatment. Each replicate used a different recording of either ambient sound or motorboat noise, and was played on a loop (resulting in six boat disturbances per hour in the Boat treatment) at the relevant nest during daylight hours (06.00–18.00). Figure 1 shows examples of mean sound-pressure and particle-acceleration levels from spectral analysis of 60 × 1 s windows (window length = 1 024, overlap = 50%) at nests. The mean ± standard error root-mean-square (RMS) sound-pressure level between 0 and 2 000 Hz across 60 s samples was 108.1 ± 0.5 dB re 1 µPa at 1 m at the 19 Ambient sites and 128.7 ± 0.2 dB re 1 µPa at 1 m at the 19 Boat sites. Further details of recordings and playbacks, including figure S1 showing the set-up of playback equipment at nests, are in the electronic supplementary material.

### (b) *Acanthochromis polyacanthus* male behaviour

Data on three key behaviours by brood-guarding males were collected. (i) Number of defensive acts (chasing/making aggressive strikes towards other fish; any potential competitors for the territory would also be potential predators of the offspring). (ii) Per cent time feeding (characteristic short or extended movements in the water column searching for and consuming plankton, and grazing on algae from the substrate). (iii) Number of instances of ‘glancing’ (where offspring eat mucus from the focal male; males do not initiate these interactions by characteristic posing, but they can choose to avoid them).

Each nest was visited by SLN every other day for 12 days between 08.00 and 16.00. Fish were given 1 min settling time to resume normal activity following the arrival of the observer, after which behaviour of the adult male was observed for 3 min at a distance of approximately 2 m from the nest. In a rigorous examination of consistency of behaviour in any fish, White *et al*. [29] showed that juvenile damselfish are consistent in behaviour over short (hours) and medium (days) timeframes, and that 3 min is sufficient to obtain a good indication of their behavioural traits. During preliminary observations on our focal species and life-stage, we found that feeding, aggression, and glancing could all be observed within 3 min. The adult male was chosen for behavioural observation as he provides a greater proportion of parental care in this species (MI McCormick 2013, personal observation) and is easily identified by his large genital papilla.

### (c) Prevalence and behaviour of other fish species

To assess whether any changes in paternal care or offspring survival were the consequence of a change in the local fish community, all fish within a 5 m radius of the nest were counted (by LP) directly after behavioural observations at each site. Fishes from the families Gobiidae and Blenniidae were excluded to avoid potentially unreliable data as species-level identification underwater was difficult. To assess whether the increased number of defensive acts by brood-guarding males was the consequence of a change in predation threat, the number of potentially predatory fish within 5 m and within 1 m of the nest was also calculated on each occasion. Potential predators were those species that had been seen previously or during this study to strike at juvenile *A. polyacanthus.* Potential predators of adults were seen only rarely. The lists of predators and other fish in the community seen surrounding nests can be found in electronic supplementary material, tables S1 and S2 respectively. The number of aggressive strikes made towards the male *A. polyacanthus* by other fish species (all of which were potential predators of the offspring) was also recorded during the behavioural observation period on *A. polyacanthus* males.

### (d) *Acanthochromis polyacanthus* offspring growth and survival

Three *A. polyacanthus* juveniles from each of the focal nests were removed for measurement by hand net at the beginning of the acoustic-exposure period. It was not possible to collect juveniles from one of the Ambient nests at day 0 due to the morphology of their coral shelter. Removals on day 0 represented between 1.2% and 7.3% of broods from different nests; the percentage removed did not differ significantly between sound treatments (Mann–Whitney test: *U* = 150, *N*_Ambient_ = 18, *N*_Boat_ = 19, *p* = 0.523). Removed juveniles were not returned to the nest after measurements were taken. Twenty more juveniles were removed for measurement at the end of the acoustic-exposure period (i.e. end of day 12) from those nests where broods had survived. Each removed fish was weighed (wet mass) and measured for standard length and body width (cross-sectional perimeter at the cloaca, and therefore not influenced by gut fullness, perpendicular to the line from the tip of the mouth to the middle of the tail used for standard length). Body width is a measure of muscular development; shape was measured as the ratio of body width to standard length. Survival was measured by whether any offspring remained at the nest at the end of the experiment.

### (e) Statistical analysis

General linear mixed-effects models (LMMs) fitted by maximum likelihood (Laplace approximation) were used (after log transformation to meet the assumption of normality where necessary), to test for the effects of sound treatment and number of days of sound exposure (including a possible interaction) on male behaviour, while controlling for the random effects of nest and time of day. Number of aggressive strikes were also included in the model of feeding behaviour to test whether aggression affected time allocated to feeding. Glancing was split into a binomial generalized linear mixed-effects model (GLMM) to test for whether glancing occurred or not, and a GLMM with Poisson errors for counts of glancing when it did occur. Results of interaction terms are presented only if significant. See the electronic supplementary material for further details of how these mixed-effects models were used. At two Boat nests, offspring survival was zero before the first parental behaviour observations could take place on day 2, thus these nests were not included in the analysis of paternal-care behaviour.

To examine differences in fish communities surrounding nests, a permutation-based, non-parametric multivariate analysis of similarity (ANOSIM) using the software PRIMER (Plymouth Routines in Multivariate Ecological Research v. 6.1.13; PRIMER-E Ltd, Plymouth Marine Laboratory, Plymouth, UK [30]) was conducted. Further details of this method can be found in the electronic supplementary material. The mean number of predatory fish within 1 m and 5 m of each of the focal nests in the two treatments were compared using Mann–Whitney *U* tests, as was the mean number of strikes made by other fish towards the *A. polyacanthus* brood-guarding male.

The number of nests where there was complete brood mortality was compared between sound treatments using a Fisher's exact test. The initial size, shape, and mass of juveniles where complete brood mortality occurred was compared with other nests using Mann–Whitney *U* tests. The changes in size, shape, and mass of *A. polyacanthus* offspring from day 0 to day 12 were compared between Ambient and Boat nests using Mann–Whitney *U* tests. *N* was determined by the number of nests where data could be collected at day 12 (i.e. not if offspring survival was zero).

## 3. Results

### (a) *Acanthochromis polyacanthus* paternal care behaviour

**Figure 2.**
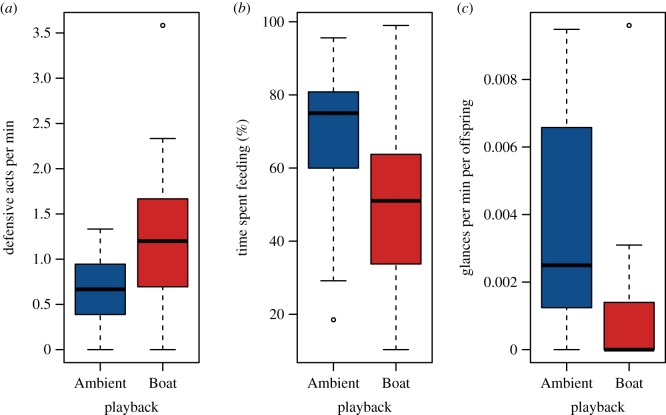
Behavioural responses to playback of motorboat noise compared with playback of ambient sound: (*a*) brood-guarding males made more defensive acts per min; (*b*) males spent less time feeding; (*c*) glancing behaviour was rarer. Boxes represent interquartile ranges and lines within boxes represent the median across 19 Ambient and 17 Boat nests. Whiskers represent ±1.5 × interquartile range. Open circles denote any data points that fall outside of the range of the whiskers. *N* determined by number of nests, data within nests averaged over duration of exposure, two Boat nests suffered complete mortality prior to first observation. (Online version in colour.)

There was a significant effect of sound treatment on defensive acts made by brood-guarding *A. polyacanthus* males (LMM: 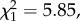 *p* = 0.016; male ID: variance = 0.18, s.d. = 0.42; time of day: variance = 0, s.d. = 0); there was no significant effect of number of days of sound exposure (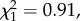 *p* = 0.340). Boat treatment males made on average twice as many defensive acts (chasing/making aggressive strikes) at other fish compared to males exposed to ambient-sound playback (figure 2*a*). Males at Boat nests also spent 25% less time feeding (displaying characteristic movements in the water column searching for and consuming plankton, or algae from the substrate) than those at Ambient nests (LMM: 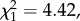 *p* = 0.036; male ID: variance = 414.87, s.d. = 20.37; time of day: variance = 11.47, s.d. = 3.39; figure 2*b*). Time spent feeding also increased with number of days of sound exposure (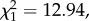 *p* < 0.001) and decreased with increasing number of aggressive strikes made by males (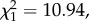 *p* < 0.001). Whether offspring glancing (eating mucus from the focal parent) occurred was not significantly affected by sound treatment (GLMM: 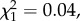 *p* = 0.848; male ID: variance = 0.07, s.d. = 0.26; time of day: variance = 0, s.d. = 0) nor by number of days of sound exposure (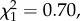 *p* = 0.403). In cases where offspring glancing did occur, it did so three times less often at nests exposed to motorboat-noise playback compared to those exposed to ambient-sound playback (GLMM: 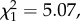 *p* = 0.024; male ID: variance = 0, s.d. = 0; time of day: variance = 0.16, s.d. = 0.40; figure 2*c*); there was a non-significant trend for a positive effect of number of days of sound exposure (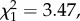 *p* = 0.063).

### (b) Prevalence and behaviour of other fish species

The increased number of defensive acts by *A. polyacanthus* brood-guarding males exposed to motorboat-noise playback compared to ambient-sound playback did not appear to be the consequence of a change in the local fish community, because there was no observed effect of sound treatment on community composition surrounding *A. polyacanthus* nest sites (ANOSIM: *R* = −0.022, *p* = 0.632; all pairwise comparisons, *p* > 0.90). Moreover, the increased number of defensive acts by brood-guarding males did not appear to be the consequence of a change in predation threat because there was no significant difference between sound treatments in the number of predatory fish within 1 m (Mann–Whitney test: *U* = 98.5, *N*_Boat_ = 14, *N*_Ambient_ = 15, *p* = 0.795) or 5 m (*U* = 92, *N*_Boat_ = 14, *N*_Ambient_ = 15, *p* = 0.582) of the focal nest. The increased number of defensive acts by brood-guarding males also did not appear to be the consequence of a change in predatory attacks, because there was no significant difference between sound treatments in the number of attacks made by other fish towards the focal *A. polyacanthus* male (*U* = 162.5, *N*_Boat_ = 18, *N*_Ambient_ = 19, *p* = 0.729).

### (c) *Acanthochromis polyacanthus* offspring growth and survival

Complete mortality of broods (survival = 0) was significantly more likely in the Boat treatment (six of 19 nests) compared to the Ambient treatment (zero of 19 nests; Fisher's exact test: *p* = 0.020). A significant difference between treatments was still apparent if the two nests suffering complete mortality in the first two days of motorboat-noise playback were removed from the analysis (*p* = 0.040). The offspring at nests that suffered 100% mortality (*N* = 6) were not significantly different in initial size (Mann–Whitney test: *U* = 74, *p* = 0.511), shape (*U* = 79, *p* = 0.664), or mass (*U* = 71, *p* = 0.432) compared with other nests (*N* = 31). At nests that did not suffer complete brood mortality and for which data were available (18 Ambient and 13 Boat nests), there was no significant effect of sound treatment on the change in juvenile fish size (Mann–Whitney test: *U* = 41, *p* = 0.262), shape (*U* = 67, *p* = 0.601), or mass (*U* = 52, *p* = 0.516).

## 4. Discussion

Defensive and feeding behaviour of *A. polyacanthus* brood-guarding males, male–offspring interactions, and survival of young were all affected by playback of motorboat noise compared to ambient-sound playback. We found no evidence of changes in tolerance, habituation, or sensitization to motorboat-noise exposure over the duration of our 12-day study (cf. [18,31]). Impacts of noise on parental-care behaviour have been shown previously [9,15,16,32]. However, our study also provides experimental evidence of an impact of anthropogenic noise on survival in free-ranging wild animals: motorboat-noise playback resulted in complete brood mortality not seen in ambient-sound playback conditions, although there was no significant difference between sound treatments in offspring growth or shape at surviving nests during our study.

Heightened stress may have caused the higher levels of aggression and chasing of other fish by *A. polyacanthus* brood-guarding males exposed to motorboat-noise playback [33]. Alternatively, stress may have caused distraction or distraction could have occurred without stress, resulting in decision-making errors [34,35]. Distraction could have led males in our study to chase and attack other fish inappropriately when exposed to motorboat-noise playback; for example, chasing fish that were not a predatory threat or chasing threatening fish less efficiently. Our findings that predator presence did not increase, but that offspring survival decreased despite increased parental acts of defence, suggests that parental-care behaviour became less efficient. One consequence of the increased defensive behaviour is reduced time spent by *A. polyacanthus* males on feeding. Motorboat-noise playback may also have impacted foraging directly, as has been seen in various other species [7,36,37]. A reduction in the acquisition of resources combined with higher energy outputs involved in nest defence would be likely to reduce body condition of parents. Measuring changes in parental condition was beyond the scope of our study but should be a focus for future work, as parental condition has previously been associated with increased mortality in offspring of *A. polyacanthus* [38].

We also found a reduction in glancing behaviour of fish exposed to motorboat-noise playback compared to those exposed to ambient-sound playback. While this is an indirect form of provisioning, with parents simply allowing young to eat their mucus, it still requires parents to be present and to undergo a cost for their offspring, as mucus is energetically expensive to produce [39]. Although the number of glances by juvenile *A. polyacanthus* may not directly determine nutritional state [27], the behaviour is likely to have adaptive functions such as the transfer of growth hormone (tiGH [40]) and building immune function [25,26]. It is possible that reduced glancing could impact growth and survival of offspring beyond the duration of our study.

A number of potential factors could have acted individually or in combination to produce the complete mortality we observed at 32% of the broods exposed to motorboat-noise playback. Parents could have abandoned or cannibalized their offspring [21,22]. Either leaving the territory permanently or stopping looking after their young while still at the territory would constitute abandoning the nest. However, we did not see a decrease in parental-care behaviour prior to nest mortality, and parents were still at the site when we returned to nests multiple times over several days after nest mortality to be sure that offspring were no longer present. Moreover, filial cannibalism is generally rare [22]; we did not observe cannibalism during behavioural observations, although we did observe predation by other fish; and the occasional observations (*N* = 4) of parental aggression towards offspring in the current study occurred in both sound treatments and not at the nests where mass mortality was recorded. Another possibility—that predation intensity increased in the presence of motorboat noise—also seems unlikely to be the explanation for our results, since greater numbers of predators were not observed in the vicinity of nests nor were attacks by other fish more likely at nests exposed to motorboat-noise playback compared to ambient-sound playback.

Instead, perhaps the most likely explanation for the greater brood failure in the Boat treatment compared to the Ambient treatment is increased risk of predation. There are two mechanisms by which predation risk could have increased. First, although we found no change in size, shape, or mass of larvae, it is possible that they suffered impaired predator-avoidance behaviour via stress and/or distraction, as has been seen in juveniles of other damselfish [14]. Second, more chasing of inappropriate species and at inappropriate times could mean males spent more time focusing attention on other fish and less time in close proximity to the nest, which may have left offspring vulnerable to predatory attack due to reduction in effectiveness of parental defence. An early descriptive study also indicated that motorboat disturbance could increase the vulnerability of fish nests: longear sunfish (*Lepomis megalotis*) were more likely to move away from their nest when a slow-moving motorboat was nearby [41]. Predators that have first located a nest are likely to return, especially if they have been successful at obtaining food from it, and so complete brood mortality could arise. This raises the question of how reproductive output over the length of a whole breeding season may be affected.

Our field study found consequences of chronic-noise exposure on the survival of juvenile *A. polyacanthus* in the wild; direct testing is needed if conclusions are to be drawn about other species. We note the important caveat that our experiment used underwater loudspeakers, which do not broadcast the full range of sounds produced by motorboats. But, it is also possible that our results are therefore conservative with respect to the full impact of motorboat noise, and recent work has found qualitatively similar fitness effects when using playbacks in tanks and real motorboats in open-water conditions [14]. Moreover, other stages of reproduction could also be affected negatively by motorboat noise: one study has indicated, for example, that spawning could be interrupted by the approach of a fast-moving powerboat [42]. Motorboats are found throughout the world wherever humans inhabit coastal areas, and our results suggest that boat noise should be considered in the management of fisheries and protected areas. In an even broader sense, anthropogenic noise is fast becoming an integral part of both marine and terrestrial ecosystems (for example, ship noise can travel for 1 000s of km underwater and more than 80% of land in the USA is within 1 km of a road [43,44]). Nest-defence behaviour is common among benthic spawning fishes and parental-care behaviour including defence of offspring is widespread in many other taxa including birds and mammals. Noise-induced increases in mortality due to impaired parental care could therefore be widespread and lead to population-level impacts.

## Supplementary Material

Motorboat noise impacts parental behaviour and offspring survival in a reef fish supplementary material

## Supplementary Material

Data

## Supplementary Material

Data

## Data accessibility

The datasets supporting this article have been uploaded as part of the electronic supplementary material.
