# Supplementary material for: Motorboat noise impacts parental behaviour and offspring survival in a reef fish
Source: Proc Biol Sci. 2017 Jun 7;284(1856):20170143. doi: 10.1098/rspb.2017.0143 (PMC5474065; doi:10.1098/rspb.2017.0143)
Supplement: Motorboat noise impacts parental behaviour and offspring survival in a reef fish supplementary material [file rspb20170143supp1.docx]

**Motorboat noise impacts parental behaviour
and offspring survival in a reef fish**

**Sophie L. Nedelec, Andrew N. Radford, Leanne Pearl, Brendan Nedelec, Mark I. McCormick, Mark G. Meekan, Stephen D. Simpson**

**Supplementary Material**

**Supplemental Experimental Procedures**

**Study site and species**

Juveniles in experimental nests had a standard length <14 mm, meaning they were <10 days post-hatching. Nests were located along the reef (2.4–3.3 m depth at mid tide) in the lagoon between Lizard, Palfrey, and South Islands and Seabird Islet in the Lizard Island group; an area covering ~1 km^2^. Nests were separated by at least 40 m to ensure independence (home ranges of parents at nests is <10 m and parents spend most of their time within 2 m (McCormick, pers. comm.). Nineteen of the 38 *A. polyacanthus* nests with offspring were allocated to the Ambient treatment and 19 were allocated to the Boat treatment; allocation alternated between treatments and nests allocated to the two sound treatments did not differ significantly in initial brood size (overall mean±SE=132±8; range=41–247; Mann Whitney test: U=158, N_Ambient_=N_Boat_=19, P=0.511).

**Acoustic recordings**

Sound pressure was recorded using a hydrophone (HiTech HTI-96-MIN with inbuilt preamplifier; sensitivity -165 dB re 1V/μPa; frequency range 2 Hz–30 kHz; calibrated by manufacturers; High Tech Inc., Gulfport MS) and a hand-held recorder (PCM-M10, 48 kHz sampling rate, Sony Corporation, Tokyo, Japan). An accelerometer (M20L; sensitivity following a curve over the frequency range 0–3 kHz; calibrated by manufacturers; Geospectrum Technologies, Dartmouth, Canada) was also available for making limited numbers of field recordings of particle motion. When using the accelerometer, both sound components were recorded via a sound card (MAYA44, ESI Audiotechnik GmbH, Leonberg, Germany) onto a laptop (Techra R840-12F, Toshiba). When recording acceleration, the x-axis of the accelerometer was oriented towards the sound source. The soundcard and laptop were calibrated using pure sine wave signals generated in SAS Lab (Avisoft, Germany), played on an MP3 player and measured with an oscilloscope. Four separate 5-min recordings of ambient sound were made in front of the Lizard Island Research Station, 20 m from the nearest coral reef where the water was 5 m deep and the hydrophone and accelerometer were at a depth of 2 m. Four different typical outboard motorboats (with 5 m long aluminium hulls and 30 hp Suzuki 2-stroke outboard motors) were also recorded in the same location for 10 min each; an approach from the shore (600 m away) over 2.5 min was followed by 5 min intense activity where the motorboat was driven around the hydrophone and accelerometer, followed by 2.5 min where the motorboat returned to the shore.

**Experimental playback**

Boat-noise playback was the experimental treatment and ambient-sound playback was necessary as a control treatment because ambient reef sound was always present during boat recordings. The Boat playback regime meant that there were six periods of nearby motorboat noise per hour. Playback soundtracks were allocated to the nests in a randomised blocked design (four soundtracks and two treatments in each block of eight nests). Playback exposure lasted 12 d as this was sufficient time to demonstrate an impact of playback on behaviour and fitness, but avoided juveniles growing to a size where they may begin to migrate from the nest (>30 d), allowing assessment of potential impacts on offspring growth.

Playbacks were broadcast from underwater loudspeakers (UW-30, frequency response 0.1–10 kHz, University Sound, Columbus, USA) connected to MP3 players (Sansa Clip+, SanDisk, Milpitas, CA, USA) via a 40 W amplifier (Kemo, Langen Germany). MP3 players, amplifiers and 12 V batteries were housed in waterproof boxes (Peli1200, Peli products, Barcelona, Spain) on the seabed. Loudspeakers were fixed by two metal poles at 1 m pointing towards a nest (Fig. S1). Occasional research station motorboat activity would have occurred within 100 m of the nests, although this would be balanced between the Ambient and Boat treatments as trials were running in parallel. Visual disturbance by motorboats is unlikely as visibility in the lagoon rarely exceeds 10 m. Our own research boat approached the study site slowly and anchored away from nests (>20 m) prior to observation periods.

Sound pressure and particle acceleration were recorded (using the recording equipment described above) during playback at treatment sites 1 m away from the speaker in the area of the water column that the young fish tended to use. Sound-pressure playbacks were measured at all 19 Ambient and 19 Boat sites but, due to accelerometer availability, it was only possible to measure particle motion at one site for each treatment. Illustrative power spectral densities are provided in Fig. 1 in the main paper. Other damselfish are known to hear frequencies up to around 1200 Hz [1]; we present acoustic data up to 2000 Hz as the hearing abilities of the focal species are currently unknown. Acoustic analyses were performed in MATLAB v2010a following the method described in [2]. The mean ± standard error root-mean-square (RMS) sound-pressure level between 0 and 2000 Hz across 60 s samples was 108.1±0.5 dB re 1 μPa at 1 m at the 19 Ambient sites and 128.7±0.2 dB re 1 μPa at 1 m at the 19 Boat sites. Playbacks of motorboat noise could not be heard above local ambient sound from other nest sites (verified with the hydrophone described above).

**Behavioural observations**

The observer for the study (SLN) was initially trained in the standardised quantification of the three behaviours by a very experienced fish observer (MIM), who has conducted 100s of hours of behavioural observations on *A. polyacanthus* and trained numerous others in this regard. Videos were then made of 12 active *A. polyacanthus* nests that were not used in the experiment. After two videos were used to explain the behaviours, the repeatability of the behavioural scoring of defensive acts and feeding was tested by comparing scores by SLN with those from two other experienced observers who scored the videos independently. There were high levels of repeatability between observers for both defensive acts (intra-class correlation: 0.901, 95% confidence interval (CI): 0.744–0.972) and feeding (0.853; 95% CI: 0.637–0.957). Since glancing could not easily be observed from the video footage and the focal fish sometimes disappeared from the field of view, a decision was made to collect data in the experiment in real-time. A snorkeler has a wide-scale view (for defensive acts) whilst also being able to collect fine-scale accurate data (on feeding and glancing); instances where the focal fish was out of sight during the observation period were also greatly reduced compared to video recordings.

At Boat sites, observations were only made during playback of motorboat noise (as playbacks were intermittent; see above). All three relevant behaviours of the brood-guarding male were recorded during observations. Rare occasions where the focal fish briefly entered a cave or chased an intruder out of sight would not have affected the behavioural scoring as they would not be chasing, feeding or glancing inside a cave and if out of sight because of chasing an intruder the behaviour (chasing) was already known.

**Fish community**

Table S1: Table of predators observed within 5 m of *Acanthochromis polyacanthus* nests:

| *Scolopsis bilineatus* |
| --- |
| *Acanthochromis polyacanthus* |
| *Pomacentrus moluccensis* |
| *Pomacentrus wardi* |
| *Pomacentrus sp.* |
| *Pomacentrus philippinus* |
| *Pomacentrus grammorhynchus* |
| *Neoglyphidodon melas* |
| *Thalassoma lunare* |
| *Abudefduf sexfasciatus* |
| *Amblyglyphidodon curacao* |
| *Neoniphon sammara* |
| *Myripristis berndti* |
| *Plectorhinchus chrysotaenia* |
| *Plectorhinchus lineatus* |
| *Lutjanus russellii* |
| *Lethrinus obsoletus* |
| *Lethrinus argentimaculatus* |
| *Octopoda* |
| *Cephalopholis argus* |
| *Cephalopholis boenak* |
| *Cephalopholis microprion* |

Table S2: Table of fish that were not predators in the community observed within 5 m of *Acanthochromis polyacanthus* nests:

| *Neopomacentrus cyanomos* |
| --- |
| *Stegastes fasciolatus* |
| *Chromis viridis* |
| *Chromis atripectoralis* |
| *Dichistodus perspicillatus* |
| *Dischistodus melanotus* |
| *Dischistodus pseudochrysopoecilus* |
| *Hemingymnus melapterus* |
| *Labridae sp.* |
| *Cheilinus chlorourus* |
| *Stegastes apicalis* |
| *Scaridae sp.* |
| *Chaetodon sp.* |
| *Chaetodon eureofasciatus* |
| *Chaetodon ephippium* |
| *Chaetodon vagabundus* |
| *Chaetodon auriga* |
| *Chaetodon aureofasciatus* |
| *Chaetodon lunulatus* |
| *Chaetodon lunula* |
| *Chaetodon plebeius* |
| *Chaetodon melannotus* |
| *Acanthuridae sp.* |
| *Caesio teres* |
| *Pterocaesio marri* |
| *Premnas biaculeatus* |
| *Amphiprion melanopus* |
| *Tetraodontidae* |
| *Pomacanthus sexstriatus* |
| *Balistidae* |
| *Apogon angustatus* |
| *Apogon properupta* |
| *Pomacentrus amboinensis* |
| *Pomacentrus simsiang* |
| *Plectropomus leopardus* |
| *Chrysiptera cyanea* |
| *Dascyllus aruanus* |
| *Epinephelus hexagonatus* |
| *Siganus doliatus* |
| *Siganus puelles* |
| *Teuthida* |
| *Zebrasoma veliferum* |
| *Mulloidichthys minicus* |
| *Lutjanus quinquelineatus* |
| *Dascyllus reticulatus* |
| *Mullidae sp.* |
| *Ostracion cubicus* |
| *Dasyatis kuhlii* |
| *Labroides dimidiatu* |
| *Corythoichthys sp.* |
| *Chaetodon auriga* |
| *Cephalopholis microprion* |
| *Apogon properupta* |
| *Chromis leucura* |
| *Cheilio Inermis* |
| *Meiacanthus grammistes* |
| *Sargocentron spiniferum* |

**Statistical methods**

General linear mixed effects models

Any effects such as slight water quality variations between nests were controlled for by the statistical models. The variance caused by and standard deviation of the variance for each random effect are presented alongside the results of models. To establish the best-fitting model, terms were eliminated one by one from a maximal model. Simplified models were compared with more complex ones using maximum likelihood ratio tests that employ chi-square statistics to establish whether a simpler model is significantly worse at explaining the data than a more complex one. If a simpler model is not significantly worse when a term is removed, the simpler model is deemed better and thus the term is dropped. If a simpler model is significantly worse, the term is maintained in the model [3]. The degrees of freedom from maximum likelihood tests presented in the Results of the main paper are the difference between the degrees of freedom of the simpler and the more complex models. All potential interactions of fixed effects were examined and are only presented where their exclusion from the model made the model significantly worse at explaining the data at the level p < 0.10.

ANOSIM methods: A frequency matrix (species by nest) was created, the data were log-transformed to reduce the influence of very abundant species, and Bray-Curtis similarity coefficients between pairs of nests were computed [4]. The ANOSIM procedure was carried out on the similarity matrix. ANOSIM generates an R statistic, which varies between 0 (similarities within and between treatments are the same) and 1 (all samples within treatments are more similar to each other than to any sample across treatments) and is tested for difference from zero with a permutation test (in this study, N=999 permutations). A one-way ANOSIM was used to compare fish communities among the two sound treatment types.

**
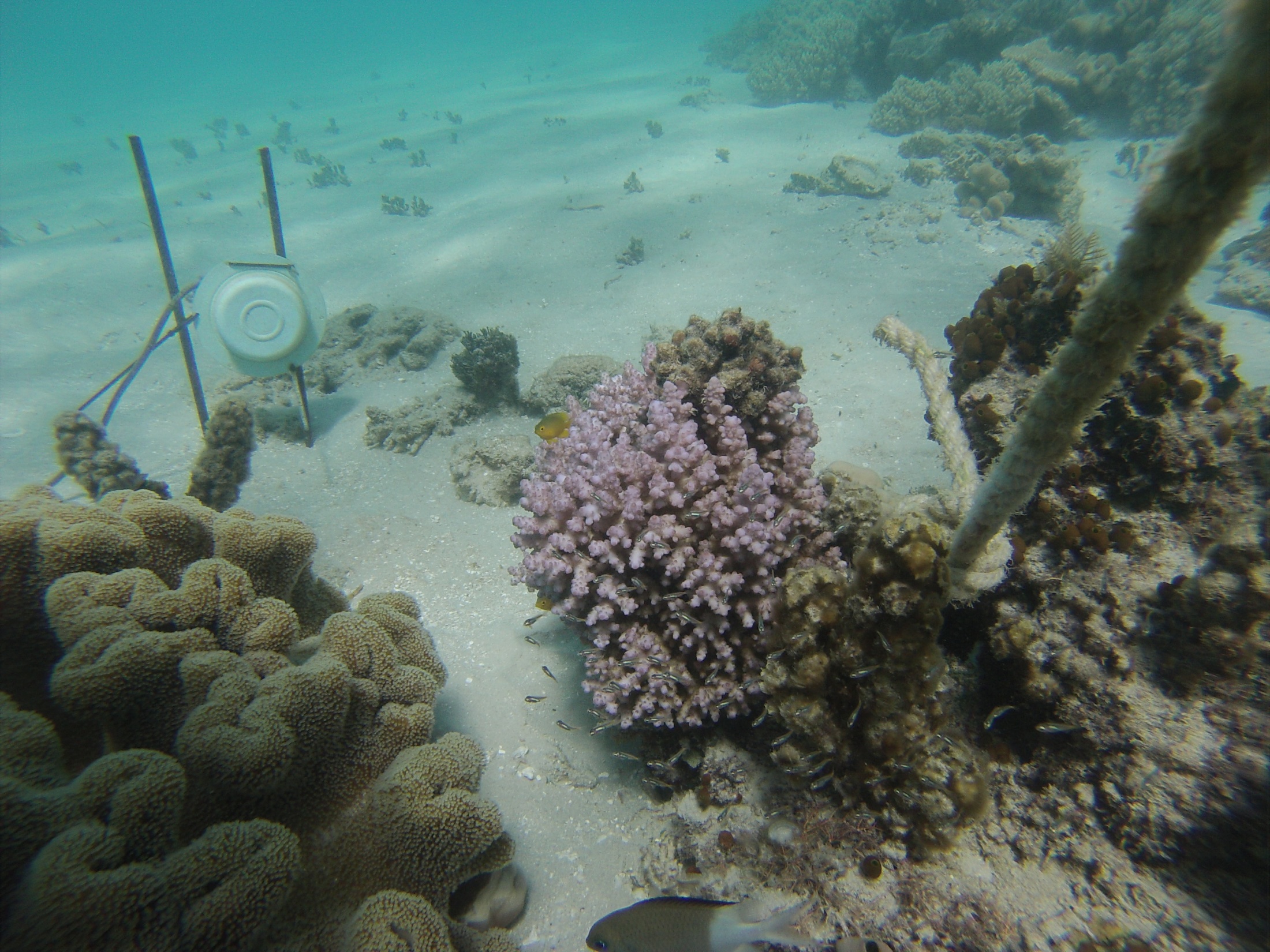
**

**Figure S1**

A photograph of one of the 38 experimental nests to illustrate the set-up; a male parent spiny chromis (middle front) can be seen guarding a brood of offspring (swimming nearby). The underwater loudspeaker used to expose this nest to the experimental playback can be seen attached to two vertical poles. Photo credit: Sophie Nedelec.

**References**

1. Ladich F, Fay R (2013) Auditory evoked potential audiometry in fish. *Rev. Fish Biol. Fisher.* **23,** 317–364. (doi: :10.1007/s11160-012-9297-z)
2. Nedelec SL, Radford AN, Simpson SD, Nedelec B, Lecchini D, Mills SC. (2014) Anthropogenic noise playback impairs embryonic development and increases mortality in a marine invertebrate. *Sci. Rep.* **4,** 5891. (doi: 10.1038/srep05891)
3. Meyer K. (1991) Estimating variances and covariances for multivariate animal-models by restricted maximum-likelihood. *Genet. Sel. Evol.* **23**, 67–83.
4. Clarke KR, Warwick RM (2001) *Change in Marine Communities: An Approach to Statistical Analysis and Interpretation*. 2nd edn. Plymouth Marine Laboratory, Plymouth, UK.
